# Accelerated epigenetic ageing after burn injury

**DOI:** 10.1007/s11357-024-01433-4

**Published:** 2025-01-16

**Authors:** Jack Sullivan, Thomas Nicholson, Jon Hazeldine, Naiem Moiemen, Janet M. Lord

**Affiliations:** 1 Inflammation and Ageing, University of Birmingham, Birmingham, UK; 2 Scar Free Foundation Centre for Conflict Wound Research, University Hospital Birmingham, Birmingham, UK; 3 NIHR Surgical Reconstruction and Microbiology Research Centre, University Hospital Birmingham and University of Birmingham, Birmingham, UK; 4 NIHR Sarcopenia and Multimorbidity Research Centre, University Hospital Birmingham and University of Birmingham, Birmingham, UK; 5 Burns Research Centre, University Hospital Birmingham, Birmingham, UK

**Keywords:** Burn injury, Epigenetic clock, Ageing, DNA methylation

## Abstract

Individuals who suffer a major burn injury are at higher risk of developing a range of age-associated diseases prematurely leading to an increase in mortality in adult and juvenile burn injury survivors. One possible explanation is that injury is accelerating the biological ageing process. To test this hypothesis, we analysed DNA methylation in peripheral blood mononuclear cells from adult burn-injured patients (> 5%TBSA) upon admission to hospital and 6 months later, to calculate an epigenetic clock value which can be used to determine biological age. Fifty-three burn-injured participants (mean age 45.43 years, 49 male, mean TBSA 37.65%) were recruited at admission and 34 again 6 months post injury (mean age 40.4 years, 34 male, mean TBSA 30.91%). Twenty-nine healthy controls (mean age 43.69 years, 24 male) were also recruited. Epigenetic age acceleration at admission by PhenoAge was + 7.2 years (*P* = 8.31e-5) but by month 6 was not significantly different from healthy controls. PCGrimAge acceleration was + 9.23 years at admission (*P* = 5.79e-11) and remained 4.18 years higher than in controls by month 6 (*P* = 2.64e-6). At admission, the burn-injured participants had a Dunedin PACE of ageing score 31.65% higher than the control group (*P* = 2.14e-12), the equivalent of + 115 days per year of biological ageing. Six months post injury the Dunedin PACE of ageing remained significantly higher (+ 11.36%, 41 days/year) than in the control group (*P* = 3.99e-5). No differences were seen using the Horvath and Hannum clocks. Enrichment analysis revealed that key pathways enriched with burn injury related to immune function, activation, and inflammation. The results reveal that epigenetic age, specifically the PACE of ageing and PCGrimAge, was accelerated in burn-injured adults at admission, with some return towards control values by 6 months. That these two clocks are built upon morbidity outcomes suggests that the injury is invoking a biological response that increases the risk of disease. Burn injury in adults induces epigenetic changes suggestive of an acceleration of the ageing process, which may contribute to the increased morbidity and mortality in these patients.

## Introduction

More than five million people die following a major injury each year, with this being the leading cause of death in those aged 15–29 years globally [1]. This high death toll is surpassed by an even greater number of patients who survive and go on to live with the long-term health consequences of critical injuries. These include long-term disability, chronic disease, and a shortened lifespan. For example, individuals who suffer a major burn injury are at a higher risk of developing a range of age-associated diseases prematurely, including cardiovascular disease [2, 3] and type II diabetes [4]. This increased morbidity ultimately leads to an increase in mortality risk in both adult [5] and juvenile burn injury survivors [6]. Poor health outcomes are also observed in other types of injury. For example, people who sustained a workplace injury were found to have a higher incidence of diseases such as arthritis and hypertension compared to their non-injured peers [7], likewise, those who sustain traumatic brain injuries exhibit higher rates of mortality and co-morbidities such as neurological and cardiovascular disease [8, 9].

Currently, it is unclear why survivors of traumatic injuries are at a greater risk of premature death and the development of many diseases that are commonly associated with advancing age. One hypothesis is that traumatic injury accelerates the rate of biological ageing [10]. Until recently, it was difficult to test this hypothesis since there were no accurate measures of how quickly an individual was ageing. However, there are now multiple biomarkers of the ageing process, which include proteomics [11], metabolomics [12], and epigenetic-based ageing clocks, the latter being one of the earliest developed and most widely used biomarkers [13].

Epigenetic clocks are based on methylation at specific sites in DNA, with DNA methylation correlating strongly with chronological age, the degree of deviation from this association being associated with both morbidity and mortality [14–16]. Studies of military veterans [17] and civilians [18] have both reported epigenetic ages higher than chronological age following traumatic stress. However, there are currently no reports of the impact of burn injury on DNA methylation age. Recently, additional clocks have been generated that focus on the pace of ageing in an individual [19]. The DunedinPACE of ageing clock has the advantage of being developed using longitudinal data over a period of years from the same cohort, incorporating clinical characteristics which change during the ageing process. This allows the DundedinPACE clock to show the “rate” rather than the “state” of ageing.

In this study, we aimed to assess the impact of burn injury upon epigenetic age and to investigate the genetic responses to injury that may be affecting any accelerated ageing seen.

## Methods

### Participants

Samples from burns patients enrolled into the prospective observational Scientific Investigation Following Thermal Injury (SIFTI)−1 and SIFTI-2 studies were analysed in this study. Blood samples were collected from participants at the first available opportunity following hospital admission and at 6 months post injury. “Admission” samples were collected within 24 h of admission to hospital (*n* = 44) except for 6 samples which were collected within 48 h and 3 which were collected within 72 h. This variation in sample collection time is due in part to a delay in sampling some of the most severely injured participants. Participants were recruited if they were aged ≥ 16 and had burns of ≥ 5% total body surface area. Inclusion and exclusion criteria as well as study protocols have been reported previously [20, 21]. Ethical approval for the SIFTI-1 study was granted by a UK NHS research ethics committee (reference 12/EM/0432). The SIFTI-2 study received ethical approval from the West Midlands, Coventry, and Warwickshire Research Ethics Committee (REC reference: 16/ WM/0217). Additional healthy age and sex-matched controls were also recruited under the ethical approval of the local ethics committee at the University of Birmingham (ERN_12–1184 & ERN_19–0831).

### DNA extraction and processing

Peripheral blood mononuclear cells (PBMCs) were isolated from whole blood using Ficoll-Paque density centrifugation (GE Healthcare, IL, USA) and frozen at − 80 °C until analysed. DNA was extracted from PBMCs using the Qiagen DNeasy Blood & Tissue Kit (Qiagen, Manchester, UK). DNA Integrity was assessed using the Agilent 2200 TapeStation system (Agilent Technologies, CA, USA). Bisulphite conversion and array processing were performed by Diagenode SA (Belgium), and samples were run on the Infinium 450K and 850K EPIC array (Illumina, CA, USA). All data processing was performed using RStudio (Version: 2023.06.1 + 524).

### Epigenetic age estimation

For epigenetic age estimation, IDAT files were processed using minfi [22] and ENMIX [23]. No samples failed initial ENMIX QC so were excluded from DNA methylation age analysis. All samples processed were included in downstream analysis. Using ENMIX, “RELIC” dye bias correction and quantile-normalisation were performed, and low-quality and outlier probe values were excluded. Horvath [15], Hannum [14], DNAm PhenoAge [16], and DunedinPACE [19] DNA methylation age analysis was performed using ENMIX. PCGrimAge [24] was calculated using the dnaMethyAge package [25]. To adjust for chronological age as a confounding factor, epigenetic age acceleration was defined as the residual of a linear regression of the epigenetic age on chronological age [26]. The mean of the Horvath, Hannum, DNAm PhenoAge, and PCGrimAge epigenetic ages was also generated for each participant and presented as a value termed “EpiMean”, and the residual of the EpiMean against chronological age was used to establish “EpiMean acceleration”. This “EpiMean” analysis was included to account for differences between epigenetic age estimates of these different clocks [27, 28].

### Differential methylation analysis

For differential methylation analysis, IDAT files were processed using minfi [22], single sample noob normalisation was used, and snps and cross-reactive probes were removed using the maxprobes package. Differential methylation and pathway analysis was performed on methylation values (getM: mvalues) using ReactomePA [29].

### Accessibility

To ensure that all figures are readable for the greatest number of people, all colour figures were generated using the Viridis colour map R package [30], which was developed to improve the readability of visual data for those who may otherwise have issues viewing them.

### Statistical analysis

Data analysis was performed using R Studio (Version: 2023.06.1 + 524). The normality of the data was assessed using the Shapiro-Wilks test. Paired or unpaired *t*-test or Wilcoxon test was used where appropriate. *P* value adjustments for multiple comparisons were made where appropriate. Significance was set at a *P* value of < 0.05: “ns” *P* > 0.05, *P ≤ 0.05, **P ≤ 0.01, ***P ≤ 0.001, ****P ≤ 0.0001. The Spearman test was used to assess correlation.

## Results

### Participant demographics

Fifty-three burn-injured participants were sampled at admission and 34 of these at 6 months post injury. Twenty-nine healthy age-matched control samples were also collected for comparison. Participant information relevant to injury outcome and severity, including total burn surface area percentage (TBSA%) [31], revised Baux score (rBaux) [32], acute physiology and chronic health evaluation score (APACHE II) [33], sequential organ failure score (SOFA) [34], and length of hospital stay (LOS) are shown in Table 1.

**Table 1:** Cohort demographic and injury information. Data for all burn patients at admission, month 6 as well as controls are shown. *TBSA%* total burn surface area percentage, *rBaux* revised Baux score, *APACHE II* Acute physiology and chronic health evaluation score, *SOFA* sequential organ failure score, *LOS* length of stay (before discharge to outpatient). Numbers at admission; TBSA% (53), LOS (48), SOFA (44), APACHE II (41). Numbers at month 6; LOS (33), SOFA (31), APACHE II (29). Data are mean, with the value range where appropriate

Total cohort	*n*	Age	Sex (M:F)	TBSA%	rBaux	APACHE II	SOFA	LOS
Control	29	43.69	24:5	n/a	n/a	n/a	n/a	n/a
		(19.65–78)						
Admission	53	45.43	49:4	37.65	90.97	21.73	6.68	35.53
		(16.43–85.75)		(4–95)	(38.81–155)	(1–46)	(0–15)	(1–216)
Month 6	34	40.94	34:0	30.91	75.69	18	4.87	43.81
		(16.95–74.23)		(4–71)	(38.81–137.31)	(0–46)	(0–15)	(6–216)

### Epigenetic clock performance

**Fig. 1:**
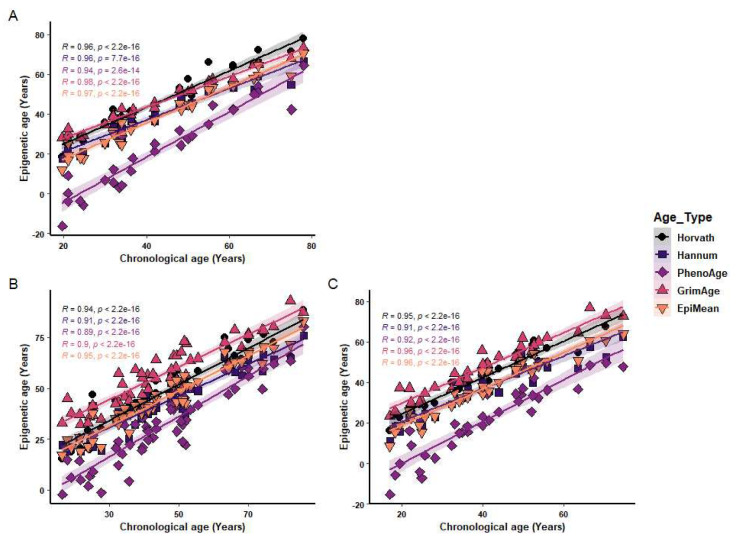
Correlation between chronological and epigenetic age. Horvath, Hannum, PhenoAge, PCGrimAge and Mean EpiAge epigenetic ages were correlated with chronological age in healthy controls (*n* = 29 (**A**)) and burn injured participants at admission (*n* = 53 (**B**)) and 6 months post injury (*n* = 34(**C**)). Spearman correlation tests were used for statistical analysis. *R* & *P* values are shown for each dataset in the top left of the plot

The epigenetic clocks examined; Horvath, Hannum, DNAm PhenoAge, PCGrimAge, and EpiMean performed as expected. All measures of epigenetic age showed a strong positive correlation with chronological age as has previously been reported previously [14–16, 35] (Fig. 1).

### Epigenetic age following burn injury

**Fig. 2:**
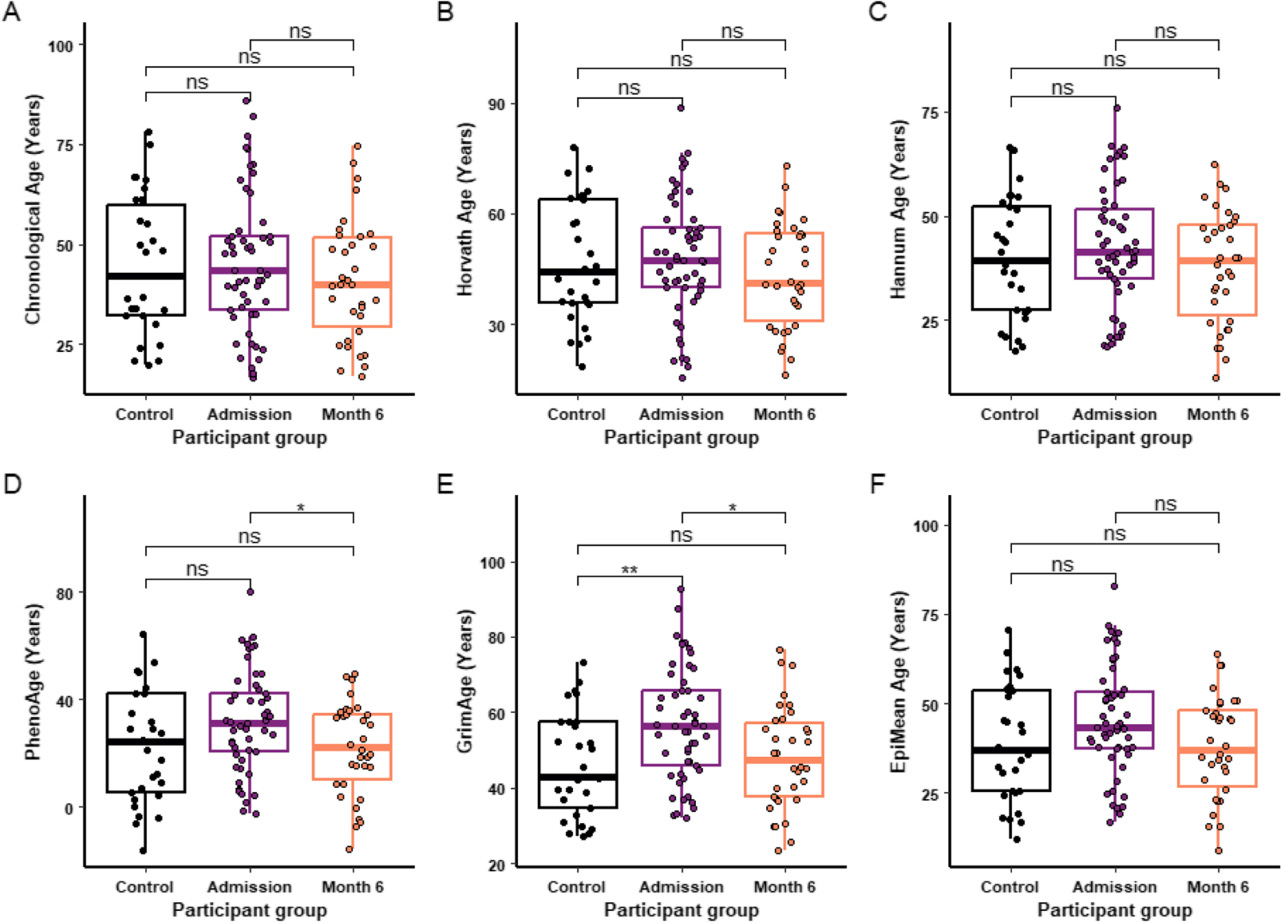
Effect of burn injury on epigenetic age. Chronological age (**A**), Phenoage (**B**), PCGrimAge (**C**), Horvath (**D**), Hannum (**E**) and Mean EpiAge (**F**) were compared in healthy controls (*n* = 29) and burn injured participants at admission (*n* = 53) and 6 months post injury (*n* = 34). For each box plot the central line is median, the bottom line is the 1st quartile (Q1), the top is the 3rd quartile (Q3). The whiskers represent calculated minimum and maximum values using the interquartile range (IQR). T tests were used for the statistical analysis of B-F, a Wilcoxon test was used for A. Significance is shown above each compared dataset, “ns” *P* > 0.05, *P ≤ 0.05, **P ≤ 0.01, ***P ≤ 0.001, ****P ≤ 0.0001

There was no significant difference in the chronological ages of the participant groups (Fig. 2A). Despite this, there were significant differences in the epigenetic ages generated. There was no significant difference between the control and injured groups according to the Horvath (Fig. 2B) and Hannum clocks (Fig. 2C). PhenoAge was significantly lower at month 6 post injury compared to admission (*P* = 0.012, Fig. 2D). PCGrimAge was higher at admission than the control group (*P* = 0.005, Fig. 2E) and month 6 (*P* = 0.013, Fig. 2E) but was no different between month 6 and control (*P* = 0.58, Fig. 2E).

**Fig. 3:**
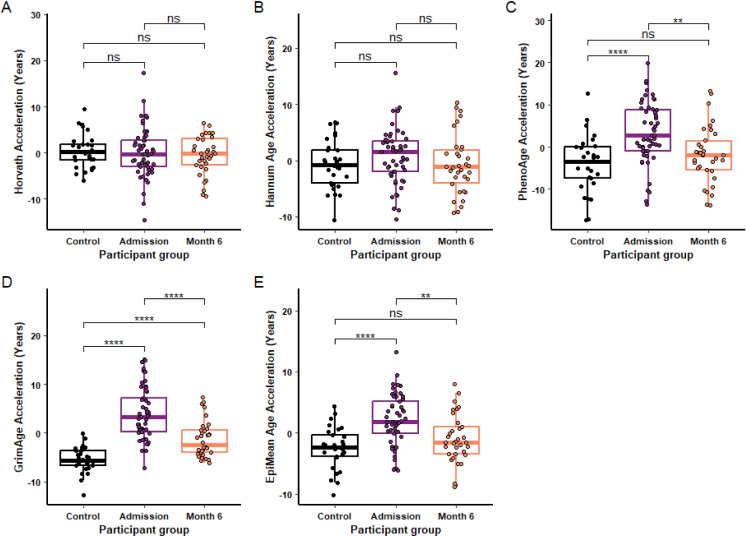
Effect of burn injury on epigenetic age acceleration. Horvath (**A**), Hannum (**B**), PhenoAge (**C**), PCGrimAge (**D**) and Mean EpiAge (**E**) epigenetic age acceleration was compared in healthy controls (*n* = 29) and burn injured participants at admission (*n* = 53) and 6 months post injury (*n* = 34) For each box plot the central line is median, the bottom line is the 1st quartile (Q1), the top is the 3rd quartile (Q3). The whiskers represent calculated minimum and maximum values using the interquartile range (IQR). T tests were used for the statistical analysis of **A**–**C** and **E**, a Wilcoxon test was used for **D**. Significance is shown above each compared dataset, “ns” *P* > 0.05, *P ≤ 0.05, **P ≤ 0.01, ***P ≤ 0.001, ****P ≤ 0.0001

There was no significant difference in epigenetic age acceleration between groups according to the Horvath (Fig. 3A) and Hannum clocks (Fig. 3B). There was however a significant difference in epigenetic age acceleration between groups according to the PhenoAge and PCGrimAge clocks, as well as the EpiMean (Fig. 3). There was significantly greater PhenoAge acceleration in the admission group of + 7.2 years (*P* = 8.31e-5) compared to healthy controls. The PhenoAge acceleration at month 6 was 5.21 years lower than at admission (*P* = 0.002) and by this time point was not significantly different from the healthy control group (p = 0.23) (Fig. 3C). PCGrimAge acceleration was + 9.23 years greater at admission compared to controls (*P* = 5.79e-11); it was lower at month 6 than at admission (*P* = 9.33e-6) and remained 4.18 years higher than in healthy controls (*P* = 2.64e-6) (Fig. 3D). The EpiMean age was higher at admission than the control group (*P* = 4.56e-5) but had returned to the control level by month 6 (*P* = 0.246) (Fig. 3E).

**Fig. 4:**
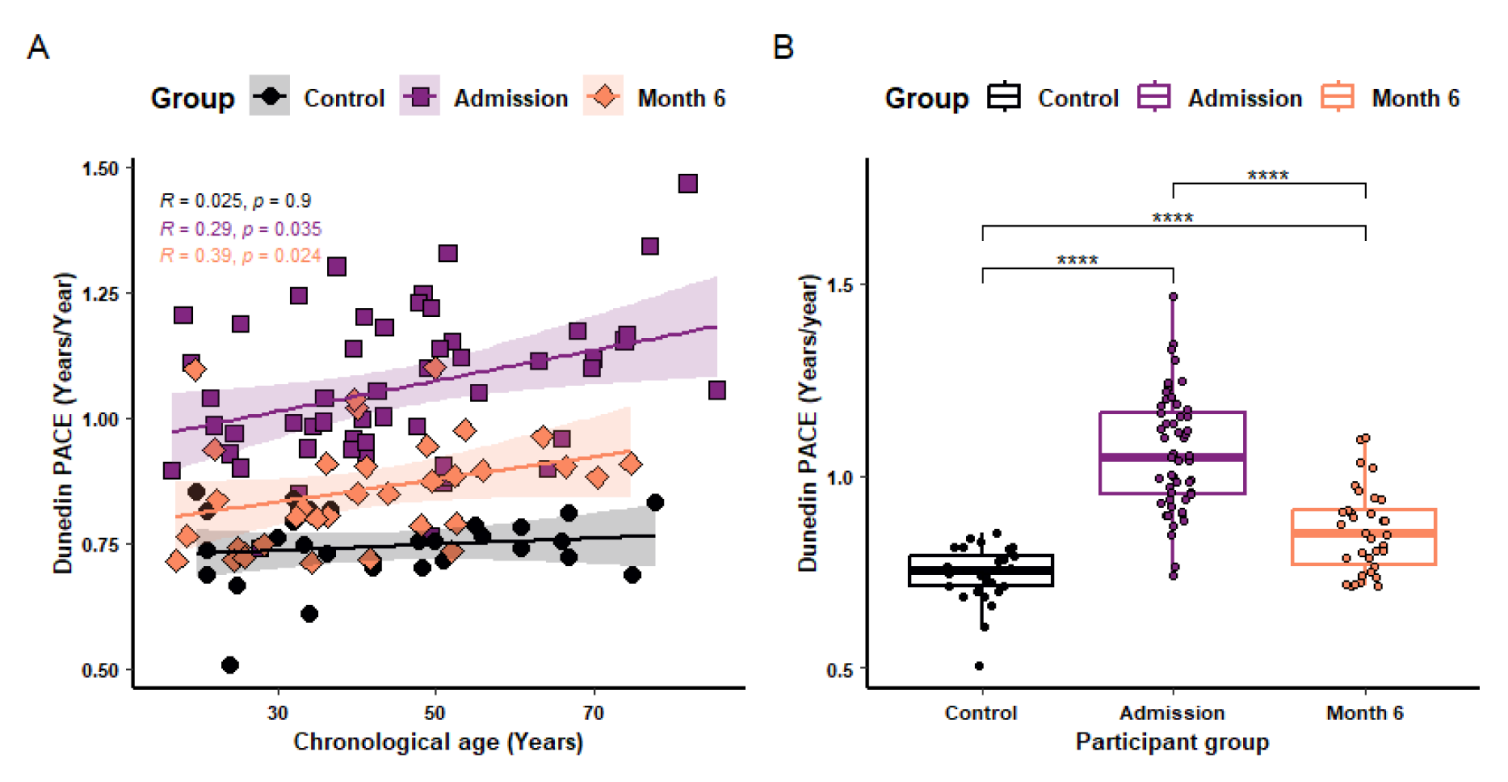
Correlation between chronological age and PACE of ageing, and the impact of burn injury. Dunedin PACE was correlated with chronological age in healthy controls (*n* = 29) and burn injured participants at admission (*n* = 53) and 6 months post injury (*n* = 34) (**A**). Spearman correlation tests were used for statistical analysis. *R* & *P* values are shown for each dataset in the top left of the plot. The Dunedin PACE for each of these groups were also compared to each other (**B**). For each box plot the central line is median, the bottom line is the 1st quartile (Q1), the top is the 3rd quartile (Q3). The whiskers represent calculated minimum and maximum values using the interquartile range (IQR). Wilcoxon tests were used for statistical analysis. Significance is shown above each compared dataset, “ns” *P* > 0.05, *P ≤ 0.05, **P ≤ 0.01, ***P ≤ 0.001, ****P ≤ 0.0001

When comparing the DunedinPACE of ageing clock between participant groups (Fig. 4), Dunedin PACE showed a positive correlation with chronological age in the burn-injured cohort at admission (*R* = 0.29, *P* = 0.035) and at month 6 post injury (*R* = 0.39, *P* = 0.024). No significant correlation was observed in the control group (Fig. 4A). At admission, the burn-injured participants had a PACE of ageing significantly (*P* = 2.14e-12) higher than the control group, 31.65% higher, the equivalent of 115 days per year. Six months post injury the participants had a PACE of ageing remained significantly higher (+ 11.36%, 41 days/year) than in the uninjured control group (*P* = 3.99e-5) (Fig. 4B).

**Fig. 5:**
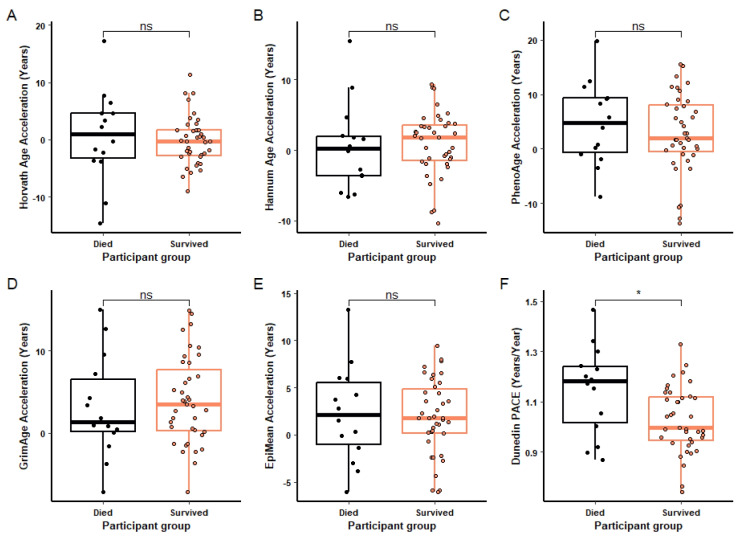
Impact of survival status on epigenetic age acceleration and PACE of ageing. Horvath (**A**), Hannum (**B**), PhenoAge (**C**), PCGrimAge (**D**) and Mean EpiAge (**E**) epigenetic ages acceleration and Dunedin PACE (F) at admission in burn injured participants who died (*n* = 14) and survived (*n* = 39) following injury. For each box plot the central line is median, the bottom line is the 1st quartile (Q1), the top is the 3rd quartile (Q3). The whiskers represent calculated minimum and maximum values using the interquartile range (IQR). *T* tests were used for the statistical analysis. Significance is shown above each compared dataset, “ns” *P* > 0.05, *P ≤ 0.05, **P ≤ 0.01, ***P ≤ 0.001, ****P ≤ 0.0001

Not all the participants sampled at admission survived their injuries. The epigenetic age acceleration and DunedinPACE of ageing of participants at admission who survived to outpatient status, and those who did not survive to outpatient status (Table 2) were compared (Fig. 5). According to the Horvath, Hannum, PhenoAge, PCGrimAge, and EpiMean clocks, there was no significant difference between survivors and those who died. However, the DunedinPACE of ageing was significantly higher in those who died following injury than those who survived (*P* = 0.035) (Fig. 5).

**Table 2:** Burn injury survival sub-group cohort information. The cohort information for all participants at admission who died following their injuries and those who survived as well as the cohort as a whole. Data shown are mean, with the value range where appropriate. *TBSA%* total burn surface area percentage, *rBaux* revised Baux score, *APACHE II* Acute physiology and chronic health evaluation score, *SOFA* sequential organ failure score, *LOS* length of stay (before discharge to outpatient). Missing data, Died; SOFA (−2), APACHE II (−2). Survived; SOFA (−7), APACHE II (−10), LOS (−5)

Cohort	*n*	Age	Sex (M:F)	TBSA%	rBaux	APACHE II	SOFA	LOS
Admission	53	45.43	49:4	37.65	90.97	21.73	6.68	35.53
		(16.43–85.75)		(4–95)	(38.81–155)	(1–46)	(0–15)	(1–216)
Died	14	54.9	10:4	41.25	109	30.08	11	14
		(25.14–85.75)		(14–79)	(79.49–155)	(4–42)	(4–15)	(1–31)
Survived	39	42	39:0	36.4	84.22	18	5.06	44.9
		(16.43–74.22)		(4–95)	(38.81–154.48)	(1–46)	(0–15)	(6–216)

### Clinical factors influencing epigenetic age in burn patients

In investigating the factors that may have influenced individual epigenetic age responses to injury (Table 3), we found a significant positive correlation between the Dunedin PACE of ageing and rBaux score only in participants at month 6 (*R* = 0.57, *P* = 0.00044), and between Horvath epigenetic age acceleration and BMI in participants at admission (*R* = 0.31, *P* = 0.027). There was no significant correlation between any other clinical markers of interest and epigenetic age acceleration or Dunedin PACE of ageing in participants at admission or 6 months post injury (Table 3).

**Table 3:** Relationship between clinical markers and epigenetic age acceleration and PACE of ageing. The epigenetic age and Pace of ageing compared to clinical markers of interest for participants at admission (n = 53) and 6 months post injury (n = 34). Statistical analysis was Spearman correlation. *rBaux* revised Baux score, *TBSA%* total burn surface area percentage, *APACHE II* Acute physiology and chronic health evaluation score, *SOFA* sequential organ failure score, *LOS* length of stay (before discharge to outpatient), *BMI* body mass index

		Variable											
		**rBaux**		**TBSA**		**APACHE II**		**SOFA**		**LOS**		**BMI**	
Clock	**Injury group**	*R*	*P* value	*R*	*P* value	*R*	*P* value	*R*	*P* value	*R*	*P* value	*R*	*P* value
Horvath acceleration	Admission	0.034	0.81	− 0.13	0.35	− 0.047	0.77	0.035	0.82	− 0.18	0.22	0.31	0.027
	Month 6	− 0.038	0.83	− 0.04	0.82	− 0.056	0.78	0.043	0.82	− 0.016	0.93	0.023	0.9
Hannum acceleration	Admission	− 0.027	0.85	− 0.001	0.99	− 0.11	0.48	0.017	0.91	0.16	0.29	0.13	0.37
	Month 6	0.16	0.36	0.15	0.41	− 0.082	0.68	0.057	0.76	0.23	0.2	− 0.16	0.36
PhenoAge acceleration	Admission	− 0.11	0.44	− 0.041	0.77	0.093	0.56	0.082	0.6	0.066	0.66	− 0.096	0.5
	Month 6	0.16	0.38	0.18	0.3	− 0.083	0.67	0.061	0.74	0.14	0.43	− 0.13	0.47
GrimAge acceleration	Admission	− 0.054	0.7	0.02	0.89	− 0.02	0.9	0.013	0.93	0.25	0.09	− 0.24	0.091
	Month 6	0.34	0.05	0.22	0.21	− 0.2	0.3	− 0.058	0.76	0.09	0.62	− 0.12	0.51
EpiMean acceleration	Admission	− 0.094	0.5	− 0.069	0.63	− 0.02	0.9	0.054	0.73	0.83	0.58	− 0.044	0.76
	Month 6	0.15	0.39	0.16	0.38	− 0.12	0.53	0.038	0.84	0.11	0.53	− 0.13	0.47
PACE	Admission	0.2	0.16	0.0053	0.97	0.17	0.3	0.26	0.088	0.0021	0.99	− 0.14	0.34
	Month 6	0.57	0.0004	0.29	0.091	− 0.008	0.97	0.043	0.82	0.27	0.13	0.13	0.45

### Differential methylation analysis

**Fig. 6:**
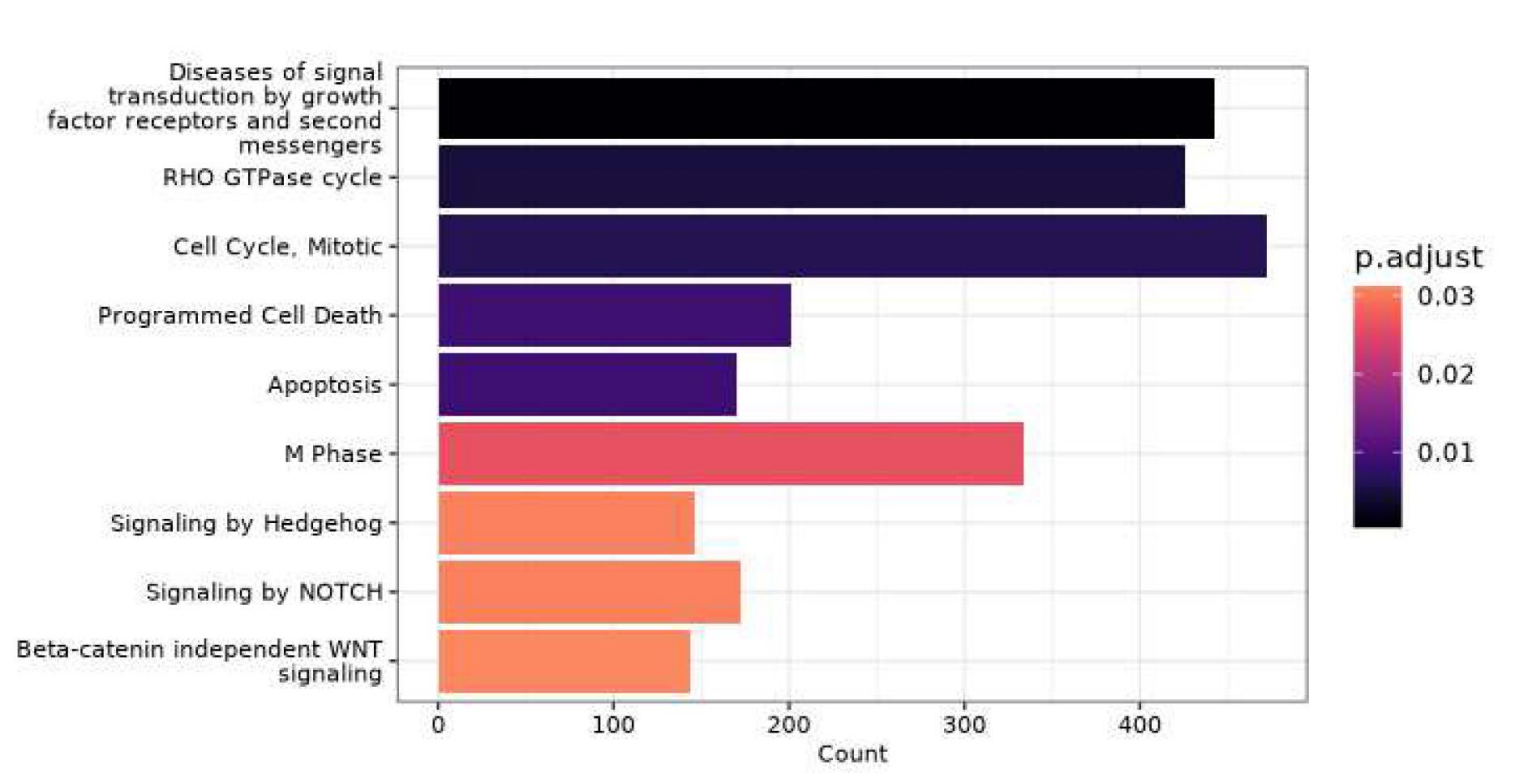
Enrichment of differentially methylated genes between participants 6 months post injury and healthy controls. Enrichment analysis was performed on the list of genes which were differentially methylated (BH adjusted *p* = < 0.05) between burn injured participants 6 months post injury (*n* = 25) and healthy controls (*n* = 24). Samples run on the 450k array were excluded from enrichment analysis due to the array not covering as wide of a range of sites as the EPIC array

The EPIC array gives greater coverage of the genome than the 450K array; thus, only the samples run on the EPIC array were used for differential methylation analysis. Enrichment analysis was performed on the significantly differently methylated genes between the control and month 6 groups to examine how burn injury affects the methylome over time. Numerous genes and pathways were differentially methylated between the control and burn-injured group 6 months post injury (adj. *P* = < 0.05) (Fig. 6). The key pathways which were enriched were related to immune function, the cell cycle, cell signalling, and inflammation. These included “Diseases of signal transduction by growth factor receptors and second messengers”, “RHO GTPase cycle”, “Cell cycle, mitotic”, “M Phase”, “Signalling by Hedgehog”, “Signalling by NOTCH”, “Beta-catenin dependent WNT signalling” as well as pathways relating to Apoptosis and “Programmed cell death”.

When comparing the methylome of the participants who died and those who survived their injuries, there were no probes which were significantly differentially methylated (adj. *P* = < 0.05).

## Discussion

There is limited research examining how physical trauma impacts the rate of epigenetic ageing. There is, however, literature examining how cumulative trauma and stress, and lifetime adversity impacts epigenetic ageing [36, 37]. It has been shown that stress, either as the result of post-traumatic stress disorder (PTSD) in veterans [38], adverse childhood experiences [39], or due to socioeconomic background and living conditions [40], all have a negative impact on the degree of epigenetic ageing. These exposures and conditions may be driving an acceleration of biological ageing by increasing the presence of factors such as chronic inflammation and increased presence of reactive oxygen species [41, 42] which have been shown to play a role in the ageing process [43]. The biological impact of burn injury is vast and long-lasting. Burn injury stimulates a large initial inflammatory response [44], shock and metabolic changes which can in turn result in organ failure [45]. Hypermetabolism and inflammation can also persist long-term [46]. This means that regardless of initial recovery from the injury itself, burns can cause long-term, negative changes in metabolic and inflammatory processes at the organismal level. Given this, it is not surprising to find that physical trauma, presented here in burn-injured participants, has a negative impact on biological ageing.

Burn-injured participants had greater epigenetic age acceleration and DunedinPACE of ageing at admission than 6 months post injury, with both being greater than in non-injured participants although not all clocks showed this. Notably the “first generation” epigenetic clocks (Horvath and Hannum) showed little relationship to injury status, which may be a result of them not having been developed using health characteristics as is the case with the PhenoAge, PCGrimAge, and DunedinPACE clocks. This difference in performance between different epigenetic clocks has been previously reported [47]. The DunedinPACE of ageing clock shows the rate rather than the state of ageing. The DunedinPACE clock, which showed significant differences between the injured and uninjured groups was developed longitudinally, using both physiological and clinical markers which change with age. The Dunedin PACE clock therefore may present the most clinically relevant insight into how the participants are ageing at the time of sampling, rather than being a record of their previous life experiences.

While previous research has shown the impact psychological trauma has on epigenetic ageing [37, 38], this is the first publication to show a link between burn injury and epigenetic ageing. Given that burn injury was associated with acceleration and PACE of epigenetic ageing, it was surprising to see that no clinical parameters of injury severity (rBaux, TBSA%, LOS etc.) were correlated with epigenetic age acceleration, excluding rBaux at admission. This may reflect either that injury severity alone is not related to the amount of age acceleration a burn injury is likely to cause or that the injury scoring systems used in this study do not fully reflect the biological stress that a burn injury may cause. The only marker of injury severity that did show a correlation with epigenetic ageing was rBaux. rBaux is generated by combining the participants’ age, TBSA%, and inhalation injury status to generate a score that reflects the severity of the injuries sustained [32]. That this score, which contains chronological age, did not correlate significantly with the PACE of ageing at admission, which did correlate with chronological age alone, suggests that injury is having a compounding effect on the PACE of ageing at admission. This is reflected in the much greater PACE of ageing at admission, compared to both controls and month 6 samples.

It is also surprising that BMI (body mass index) did not influence any of the epigenetic acceleration measures, with the exception of Horvath, as it is known to increase epigenetic age [48]. This may reflect that the injury overrides the BMI effect.

Participants who died had a greater DunedinPACE of ageing than injured participants who survived their injuries. Some epigenetic clocks, such as the GrimAge clock, have shown that accelerated epigenetic ageing is positively correlated with mortality and morbidity risk [35], supporting the data presented in this study. Other studies have shown a relationship between accelerated epigenetic ageing and the risk of sepsis and death within the critical care environment [49]. What is less clear however is which direction these causalities work. One interpretation is that serious injury is increasing the rate of biological ageing, therefore reducing resilience and increasing the risk of death, something which has previously been suggested [10]. Our data would support this as we found epigenetic age acceleration at admission for some clocks when compared to age-matched controls. The second possibility is that individuals already have an increased biological age at the time of injury and are less biologically resilient, increasing the risk of death following injury. This second hypothesis is supported in principle by a meta-analysis showing that increased frailty is associated with increased mortality following multisystem trauma [50] and by the fact that chronological age is a primary risk factor for almost every major disease [51]. Increasing chronological age is also predictive of worse outcomes in the event of traumatic injury [52], sepsis [53, 54], and burn injury [55]. Given the data presented here, it is likely that both scenarios are true, though the relatively young age of our cohort gives more credence to the former interpretation.

While some of the increases in age acceleration, for example, an increased PACE of ageing of just over a month of additional biological ageing per chronological year post injury may not initially sound like a lot, it is a cause for concern as we know that we age faster the older we get [19]. This may mean that while the age acceleration at the 6-month time point may be modest in the scope of life lasting 80 + years, it could result in a steeper ageing trajectory, where the rate of ageing increases even faster, year by year, perhaps explaining the premature incidence of age-associated diseases often seen in burn survivors.

Overall, our methylation data aligns with the description of burn injury within the literature, with changes to pathways relating to immune function and inflammation persisting over time that are known drivers of the ageing process [43]. Compared to healthy controls there were pathways with associated genes that remained differentially methylated 6 months post injury. Because the EPIC array gives greater coverage of the genome than the 450K array (around double the number of probes) only the samples run on the EPIC array were used for differential methylation analysis.

The immune response to burn injury is characterised by changes in immune cell proportions, with an initial influx of immature leukocytes and neutrophils [54]. This is often accompanied by a shift towards a proinflammatory cytokine profile, known as inflammatory response syndrome [56]. Long-term persistent chronic systemic inflammation persists after burn injury [46] and it has previously been reported that there is an increase in memory cells and altered immune cell subset proportions [57]. Differential methylation and pathway enrichment analysis revealed a significant number of pathways showing enrichment. The enriched pathways concerned the cell signalling, the cell cycle, and the immune cell function. Enriched pathways related to the cell cycle included pathways such as RHO GTPases, which regulate migration and function of innate immune cells during inflammation [58] and have been shown to play a vital role in cytokinesis, apoptosis, and T lymphocyte activation and differentiation [59]. “Cell cycle, Mitotic” and “M Phase” pathways were also enriched and may reflect the clonal expansion observed in the immune system during immune activation, as for example, is observed in the B and T lymphocytes [60, 61]. The Hedgehog, NOTCH, and WNT signalling pathways specify early patterning and organ formation by regulating spatial cell proliferation and differentiation [62]. In the context of injury, Hedgehog signalling has been shown to be upregulated in the skin, with its function being to signal to immune cells and stimulate the inflammatory stage of the healing process [63]. Specifically in immune cells, Hedgehog signalling has been associated with the proinflammatory response in macrophages, is associated with the accumulation of natural killer T cells, and increases the infiltration of regulatory T cells at the site of inflammation [64] which is an abundant post burn injury. Likewise, NOTCH signalling has been shown to modulate immune cell differentiation, signal transmission, and systemic inflammation exhibiting both pro and anti-inflammatory effects in a context-dependent manner [65]. WNT signalling was also enriched. WNT signalling has been associated with immune cell development, activation, and migration factors [66], all of which are important during wound repair, an ongoing process following major injury or surgery, factors present in our burn-injured cohort.

Burn injury has previously been shown to promote an immune ageing phenotype characterised by a blunted immune response, a reduction in naïve cells, and an increased proportions of memory cells [57], which may be accompanied by an increased proportion of senescent immune cells [67]. Given the senescent cells’ resistance to apoptosis [68], it may not be surprising to see that pathways related to cell death and apoptosis remain differentially methylated 6 months post injury when compared to the healthy controls. Burn injury is also known to have an immunosuppressive effect after the initial stages, alongside apoptosis-induced lymphopenia [69], another potential explanation for the enrichment of the “programmed cell death” and “Apoptosis” pathways.

“Diseases of signal transduction by growth factor receptors and second messengers” is a broad disease-associated pathway. This is because issues with signal transduction via receptors of messenger proteins are associated with myriad diseases, rather than one specific disease. This is due to the vital role signalling plays in organismal development, normal function, and the regulation of homeostasis, where any disruption can have significant negative effects [70]. Differential methylation of this pathway in the context of burn injury may be reflective of general changes to immune cells long-term. This is supported by studies showing a blunted immune response up to 3 years post burn injury [57].

The enriched pathways related to immune function, activation, and inflammation are factors classically associated with inflammation and ageing more generally and are often seen as a vicious cycle which drive each other [71]. It may be that burn injury is inducing the kind of low levels of chronic sterile inflammation classically seen in ageing, which in turn is further accelerating the ageing process itself, a hypothesis which has previously been proposed [10].

Unlike the comparison between month 6 and control, there was no significant differential methylation between survivors and those who died following injury. This may suggest that the differences between survivors and non-survivors are not explained or captured by the DNA methylation data presented in this study. It is possible to interpret this lack of difference as meaning that DNA methylation changes may be more subtle than we are able to distinguish in this study, or that factors occurring at a time after sample collection contributed towards their lack of survival.

One limiting factor in our study is that the burn-injured participants in this study often had co-morbidities. Mental health conditions such as depression [72] and physical attributes such as poor cardiovascular health [73] have previously been associated with epigenetic age acceleration. Due to the nature of collecting samples from individuals admitted to the hospital with a burn injury, it was not possible to exclude those with co-morbidities which may impact their biological ageing as was the case with the control cohort. Another limitation of this study is that there was no pre-injury sample, meaning the degree to which injury alters the ageing trajectory of participants over time cannot be fully determined. Therefore, it is not possible to establish if the participants’ rate of ageing in injured participants was greater than that of the controls pre-injury.

In conclusion, the results presented show that burn injury has a negative effect on biological ageing and that changes to DNA methylation which occur because of injury may persist long-term. Both findings may represent targets for improving the long-term health of burn injury survivors. Future studies could build on these findings by carrying out our small-scale clinical trials to determine if the epigenetic ageing seen in injured patients can be reversed, using interventions such as exercise [74] or pharmacological interventions [27] which have been shown to reduce ageing as measured by epigenetic clocks.

## Data Availability

Data available upon reasonable request to the corresponding author: JS.
